# Ectopic Expression of *CsKCS6* From Navel Orange Promotes the Production of Very-Long-Chain Fatty Acids (VLCFAs) and Increases the Abiotic Stress Tolerance of *Arabidopsis thaliana*

**DOI:** 10.3389/fpls.2020.564656

**Published:** 2020-10-06

**Authors:** Wenfang Guo, Qi Wu, Li Yang, Wei Hu, Dechun Liu, Yong Liu

**Affiliations:** Department of Pomology, College of Agronomy, Jiangxi Agricultural University, Nanchang, China

**Keywords:** Newhall navel orange, cuticular wax, *KCS6*, abiotic stresses, transgenic *Arabidopsis*

## Abstract

Cuticular wax is closely related to plant resistance to abiotic stress. 3-Ketoacyl-CoA synthase (KCS) catalyzes the biosynthesis of very-long-chain fatty acid (VLCFA) wax precursors. In this study, a novel *KCS* family gene was isolated from Newhall navel orange and subsequently named *CsKCS6*. The CsKCS6 protein has two main domains that belong to the thiolase-like superfamily, the FAE1-CUT1-RppA and ACP_syn_III_C domains, which exist at amino acid positions 80–368 and 384–466, respectively. *CsKCS6* was expressed in all tissues, with the highest expression detected in the stigma; in addition, the transcription of *CsKCS6* was changed in response to drought stress, salt stress and abscisic acid (ABA) treatment. Heterologous expression of *CsKCS6* in *Arabidopsis* significantly increased the amount of VLCFAs in the cuticular wax on the stems and leaves, but there were no significant changes in total wax content. Compared with that of the wild-type (WT) plants, the leaf permeability of the transgenic plants was lower. Further research showed that, compared with the WT plants, the transgenic lines experienced less water loss and ion leakage after dehydration stress, displayed increased survival under drought stress treatment and presented significantly longer root lengths and survival under salt stress treatment. Our results indicate that *CsKCS6* not only plays an important role in the synthesis of fatty acid precursors involved in wax synthesis but also enhances the tolerance of transgenic *Arabidopsis* plants to abiotic stress. Thus, the identification of *CsKSC6* could help to increase the abiotic stress tolerance of *Citrus* in future breeding programs.

## Introduction

Very-long-chain fatty acids (VLCFAs), which refer mainly to a class of fatty acids with more than 18 carbon atoms, are precursor materials for the synthesis of plant cuticular waxes (Kunst and Samuels, 2003). The biosynthesis of VLCFAs involves catalysis by the multienzyme fatty acid elongase (FAE) complex. The catalytic process involves four main steps: condensation, reduction, dehydration and re-reduction (Haslam and Kunst, 2013). 3-Ketoacyl-CoA synthase (KCS) is the rate-limiting enzyme of the condensation reaction and is a key enzyme in the fatty acid extension pathway (Rossak et al., 2001).

There are many genes that encode KCS enzymes. *FAE1*, which is expressed only in seeds, was the first KCS family gene cloned from *Arabidopsis*, and its encoded protein catalyzes the synthesis of C_20_ and C_22_ fatty acids and storage lipids (James et al., 1995). To date, 21 *KCS* genes have been annotated in the Arabidopsis genome, and they are divided into four subfamilies, *FAE1*-like, *KCS1*-like, *FDH*-like, and *CER6*, based on their amino acid sequence homology (Costaglioli et al., 2005). Subsequently, Joubès et al. (2008) divided the existing 21 Arabidopsis KCS proteins into eight subclasses based on phylogeny. Guo et al. (2015) collected KCS genes from 26 sequenced plant genomes and reconstructed the phylogenetic tree of the *KCS* family genes to reveal the molecular basis of the functional divergence of different *KCS* genes. Furthermore, three *KCS* genes, including *KCS1* (Todd et al., 1999; Lee and Suh, 2013), *FDH* (Yephremov et al., 1999; Pruitt et al., 2000) and *CER6* (Millar et al., 1999; Fiebig et al., 2000; Hooker et al., 2002), have been shown to be involved in the formation of the cuticle in Arabidopsis: it has been reported that *KCS1* is expressed in all tissues of Arabidopsis plants, and complete loss of function of *KCS1* leads to a decrease in C_26_ and C_30_ alcohol and aldehyde compounds, respectively (Lee and Suh, 2013). *FDH* is expressed mainly in the flowers and young leaves of Arabidopsis; the *FDH* gene participates in the synthesis of VLCFAs in epidermal cells and is involved in both the regulation of plant morphological structure and the response to biotic stress (Yephremov et al., 1999; Pruitt et al., 2000; Joubès et al., 2008). *KCS6*/*CER6/CUT1* is expressed in the aboveground tissues of Arabidopsis, especially the flowers and siliques. Overexpression of *KCS6*/*CER6* caused an increase in the wax content of the stem epidermis of Arabidopsis. Moreover, its functional defect mutant *cer6* accumulated a large quantity of C_24_ VLCFA derivatives, indicating that this gene is involved in the biosynthesis of C_24_ (or longer) VLCFAs (Millar et al., 1999; Fiebig et al., 2000; Hooker et al., 2002).

Cuticular wax is the first protective barrier that covers the surface of terrestrial plants and is in contact with the outside environment (Riederer and Schneider, 1990; Jenks et al., 1994). Therefore, cuticular wax plays an important role in plant resistance to various stresses (Krauss et al., 1997; Sieber et al., 2000). In recent years, many wax synthesis genes have been used to improve plant resistance to abiotic stresses. *ECERIFERUM* (*CER1*) genes are involved in the wax biosynthesis pathway. *cer1* mutants present glossy green stems and altered wax alkane biosynthesis (Bourdenx et al., 2011; Bernard et al., 2012). Overexpression of *OsGL1-2* increases the number of wax crystals of rice leaves, thereby improving the drought resistance of the plant (Islam et al., 2009). Transformation of *Arabidopsis* with *WXP1* and *WXP2* also increased the wax content of the transformants and their resistance to water stress and drought stress (Zhang et al., 2005, 2007). The apple AP2/EREBP transcription factor *MdSHINE2* provides drought resistance by regulating wax biosynthesis (Zhang et al., 2019a). Similarly, the R2R3 MYB transcription factor *MdMYB30* modulates plant resistance against pathogens by regulating cuticular wax biosynthesis (Zhang et al., 2019b). Overexpression of *BnKCS1-1*, *BnKCS1-2*, and *BnCER1-2* in *Brassica napus* increased the wax crystal density on the leaf surface, and the water loss rate decreased together with increased drought tolerance, which was enhanced in transgenic plants (Wang et al., 2020).

Citrus is a perennial woody plant species with a high degree of heterozygosity. The relationship between wax genes and citrus resistance to abiotic stress may be more complex than that of model plant species such as *Arabidopsis*. In our previous studies, we analyzed the wax structure, chemical composition, and transcript levels of wax genes in Newhall navel orange (Liu et al., 2012, 2015). No wax-related genes have been identified in citrus so far; thus, the molecular mechanism underlying wax synthesis and its effect on citrus abiotic stress resistance is still unknown. In this study, a *KCS6* homologous gene, which was named *CsKCS6*, was cloned from Newhall navel orange. The expression patterns of *CsKCS6* in different tissues and in response to different abiotic stress conditions were analyzed by quantitative real-time PCR (qRT-PCR). The important role of *CsKCS6* in citrus wax synthesis and its effects on abiotic tolerance were identified by overexpressing the gene in *Arabidopsis*. The results broadened our knowledge concerning the molecular mechanism underlying cuticular wax synthesis in citrus.

## Materials and Methods

### Plant Materials and Growth Conditions

Tissue samples of from adult trees of Newhall navel orange (*Citrus sinensis*) were collected in an orchard in Xinfeng County, Jiangxi Province, China. Young leaves of Newhall navel orange trees were collected for gene cloning, and leaves, flowers, receptacles, petals, pistils, stigmas, ovaries, flavedos, albedos, and flesh were collected from five trees per biological replicate for organ-specific expression analysis. Flavedo, albedo and flesh samples were collected from five trees per biological replicate at 60, 120, and 210 days after flowering, respectively, for analysis of expression during fruit development. Three biological replicates were used for these expression analyses.

Prior to the stress treatments, 1-year-old grafted seedlings of Newhall navel orange whose height was 30 ± 2 cm were transferred to pots that contained Hoagland’s solution in a growth chamber for 7 days to acclimate to the new conditions. The plants were then subjected to 4°C for 0, 1, 3, 6, 9, 12, and 24 h for the cold treatment. For the drought stress, salt stress and abscisic acid (ABA) treatments, we transferred the plants to Hoagland’s solution that contained 20% PEG6000, 250 mM NaCl, or 100 μM ABA, respectively, for 0, 1, 3, 6, 9, 12, and 24 h. The seedlings for each treatment were grown under a light/dark cycle of 16/8 h at 25°C. For each treatment, the leaves were sampled from 18 randomly selected seedlings at each time point, and each treatment was repeated three times for each time point.

### Cloning and Sequence Analysis of *CsKCS6*

The *KCS6* homologous cDNA sequence (Cs7g13310) from sweet orange was retrieved from the sweet orange genomic database^1^. A pair of gene-specific primers (P1) (Supplementary Table S1) based on the *KCS6* homologous cDNA sequence were designed for CDS sequence cloning. The leaves of 1-year-old grafted seedlings of Newhall navel orange were sampled to extract the total RNA. RNA extraction method refers to TaKaRa MiniBEST Plant RNA Extraction Kit (TaKaRa, Japan) instructions. The integrity of RNA was detected by 1% agarose gel electrophoresis. The total RNA yield and quality were further determined using a NanoDrop 2000 spectrophotometer (Thermo Fisher Scientific, Waltham, United States). Using the total RNA extracted above as a template, TaKaRa PrimeScript^TM^ RT reagent Kit with gDNA Eraser reverse transcription kit (TaKaRa, Japan) was used for cDNA synthesis. Bioinformatic analysis, which included a plant KCS6 homology analysis, multiple sequence alignment and a KCS6 phylogenetic analysis, was conducted using the methods of a previous report (Liu et al., 2017).

### Expression Analysis of *CsKCS6* in Navel Orange

Real-time quantitative PCR (qRT-PCR) was used for *CsKCS6* transcript analysis with the specific primer P2 (Supplementary Table S1). The citrus *ACTIN* gene was amplified with the specific primer P3 (Supplementary Table S1) to serve as a control gene for normalizing the expression between different samples. The real-time quantitative detection is done using SYBR^§^ Premix Ex Taq^TM^ kit (TaKaRa, Japan). The reactions were performed by an initial incubation at 95°C pre-denaturation for 30 s, then 95°C denaturation for 5 s, 60°C annealing for 30 s and 40 cycles. The 2^–ΔΔCT^ method was used to calculate the relative changes in gene expression. qPCR was performed in four replicates for each sample, and data are indicated as the mean ± SD (*n* = 3).

### Construction of the *CsKCS6* Expression Vector and Its Genetic Transformation Into *Arabidopsis*

The full-length CDS of the Newhall navel orange *CsKCS6* gene was cloned into a pCAMBIA1301 with CaMV35S promoter plant expression vector using the amplification-specific primer P4 (Supplementary Table S1) with *Bam*HI and *BstpI* restriction sites. A vector-containing plasmid was introduced into *Agrobacterium tumefaciens* strain LBA4404, which was subsequently transformed into *Arabidopsis* by the *Agrobacterium*-mediated floral-dip method, as described by Zhou et al. (2019). Seeds of T_0_ transgenic plants were sown in Murashige and Skoog (MS) media to which hygromycin (10 mg L^–1^) was added to screen for positive T_1_ transgenic plants based on their resistance to hygromycin, and using the leaves of resistant seedlings as templates, PCR detection was carried out using gene specific primers of P4 and P7 (Supplementary Table S1). The expression level of positive transgenic plants was further tested by qPCR amplification of *CsKCS6* and *AtACTIN* using two different primers: P5 and P6 (Supplementary Table S1). Seeds of the T_1_-positive *Arabidopsis* plants were grown on MS solid media that contained hygromycin (10 mg L^–1^) to produce T_2_ transgenic plants, which were used in subsequent experiments.

### Extraction of Cuticular Waxes and Gas Chromatography-Mass Spectrometry (GC–MS) Analysis

Approximately 100 mg of leaves and stems of 6-week-old wild-type (WT) and transgenic Arabidopsis plants were removed. The sample is gently moistened with distilled water and then the tissue surface is tenderly drained with filter paper, after which they were placed in a reaction flask. Cuticular waxes were extracted described by immersing tissues for 1 min in 2 mL of chloroform containing 10 μg of tetracosane as an internal standard. The extracts were dried under a gentle stream of nitrogen gas. The dried wax residues were derivatized by adding 20 μL of silylation reagent (BSTFA) and 20 μL of pyridine and incubated for 40 min at 70°C. The chromatographic column used in the experiment adopts a universal (30 m × 0.25 mm × 0.25 μm) hp-5MS capillary column with polydimethylsiloxane as the stationary phase. The chromatographic conditions were the inlet temperature of 280°C, the column flow rate of 2 mL/min and a constant flow rate; the Aux-2 temperature of 280°C, helium as the carrier gas, sample injection without splitting and the injection volume of 1 μL. The mass spectrometry program is set to electron impact ionization mode, energy 70 eV, mass scanning range 50 ∼ 600 amu and full scanning mode. The temperature rise process is to hold at 50°C for 1 min, raise the temperature to 170°C at 20°C/min, and hold for 2 min; then raise the temperature to 300°C at 5°C/min and hold for 8 min. The ion peaks of various wax components are detected by GC–MS (GCMS-QP2010, Shimadzu, Agilent). According to the mass spectrum library to search and determine and compare the FID peak area data, the single wax compound was quantified relative to the internal standard. Three biological replicates were used for each sample. The specific methods used were described by Chen et al. (2011) and Liu et al. (2012).

### Scanning Electron Microscopy (SEM)

Scanning electron microscopy (SEM) was used to observe the ultrastructure of the wax crystals of the stems and rosette leaves of 6-week-old *Arabidopsis*. The samples were fixed in 4% glutaraldehyde for 12 h and then rinsed with 0.1 mM phosphate buffer at a pH of 7.2, three times for 10 min each. Ethanol at concentrations of 30, 50, 70, 80, and 90% was dehydrated once for 15 min using a JFC-1100-type ion sputtering instrument for gold plating. The samples were ultimately observed with a JSM-T300 scanning electron microscope (JEOL, Tokyo, Japan) after they were freeze-dried and adhered to the sample table (Liu et al., 2012).

### Toluidine Blue (TB) Staining and Chlorophyll Content Assays

Six-week-old healthy *Arabidopsis* leaves were stained in a 0.05% TB staining solution at 25°C for 10 min. The samples were then rinsed with deionized water at least three times before being images.

Healthy leaves of transgenic plants and WT plants at 6 weeks of age were taken in equal amounts, and their chlorophyll leaching content was measured according to the method of Zhang et al. (2019a).

### Physiological Measurements of *Arabidopsis* Plants Under Drought and Salt Treatments

The water loss rate and ion leakage rate of the leaves after dehydration treatment were measured according to previously described methods (Dai et al., 2018; Xie et al., 2018).

Seeds of WT and *CsKCS6*-overexpressing *Arabidopsis* were selected and sown on MS media that were supplemented with 100 mM NaCl. Thirty seeds were sown per treatment, and the germination percentage was determined daily (Seo et al., 2009). WT and transgenic *Arabidopsis* seedlings that had grown on MS media for 7 days were transferred to fresh MS media supplemented with 100 and 200 mM NaCl and allowed to grow for 2 weeks, at which time they were imaged and their root length (≥5 mm) was measured.

WT and transgenic *Arabidopsis* that had grown on MS media for 4 weeks were transplanted into soil. After 10 days, the plants were then subjected to drought (water replenishment for 3 days after 10 days of natural drought) and salt (100/200 mM NaCl solutions were poured into the pots every 3 days, at 50 ml pot^–1^) treatments. Twenty plants were included per treatment, each of which included three biological replicates (Dai et al., 2018).

### Data Presentation and Statistical Analysis

All quantitative data are given as the means and standard errors. Statistical analyses were conducted using Student’s *t*-test and Duncan’s multiple range tests in SPSS version 22. Statistical significance was considered at the *P* < 0.01 and *P* < 0.05 level for *t*-test and at *P* < 0.05 for Duncan’s multiple range tests.

## Results

### Cloning and Sequence Analysis of *CsKCS6*

**FIGURE 1:**
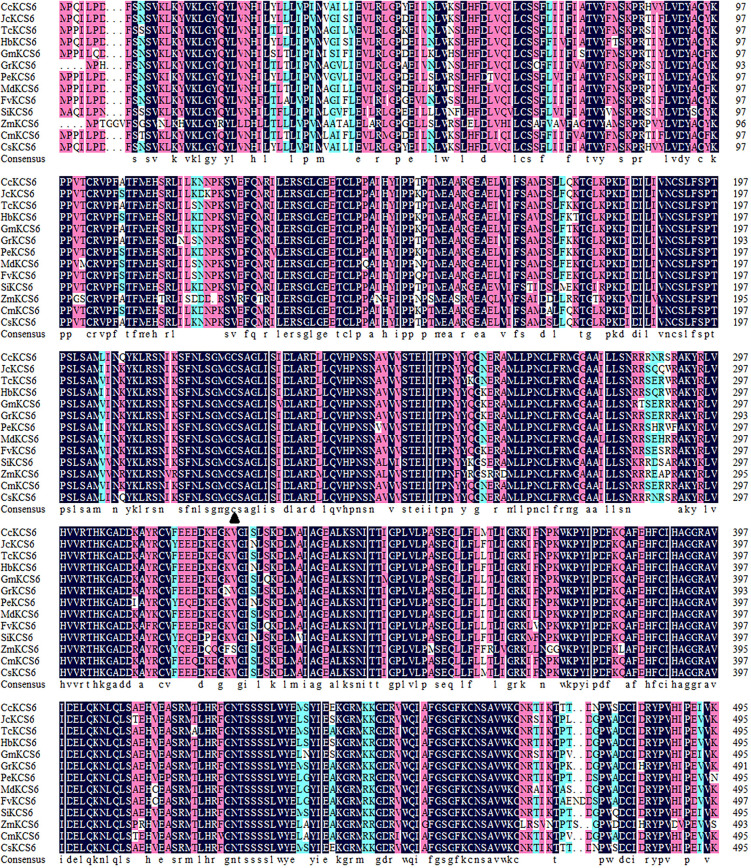
Multiple amino acid sequence alignment of CsKCs6 with its homologous proteins from different plant species, including *Citrus clementina* CcKCS6 (XP_006437664.1), *Jatropha curcas* JcKCS6 (XP_012082087.1), *Theobroma cacao* TcKCS6 (XP_007044356.2), *Hevea brasiliensis* HbKCS6 (XP_021637941.1), *Glycine max* GmKCS6 (XP_003555901.1), *Gossypium raimondii* GrKCS6 (XP_012467884.1), *Populus euphratica* PeKCS6 (XP_011007926.1), *Malus domestica* MdKCS6 (XP_008339680.2), *Fragaria vesca* subsp. *Vesca* FvKCS6 (XP_004299082.1), *Sesamum indicum* SiKCS6 (XP_011099559.1), *Zea mays* ZmKCS6 (NP_001310469.1), *Cucumis melo* CmKCS6 (XP_008446598.1), *Arabidopsis thaliana* AtKCS6 (AAM65060.1), *Ginkgo biloba* GbKCS6 (AAY52458.1), and *Citrus sinensis* CsKCS6. The Cys amino acid (c) site is marked with solid triangle (▲).

**FIGURE 2:**
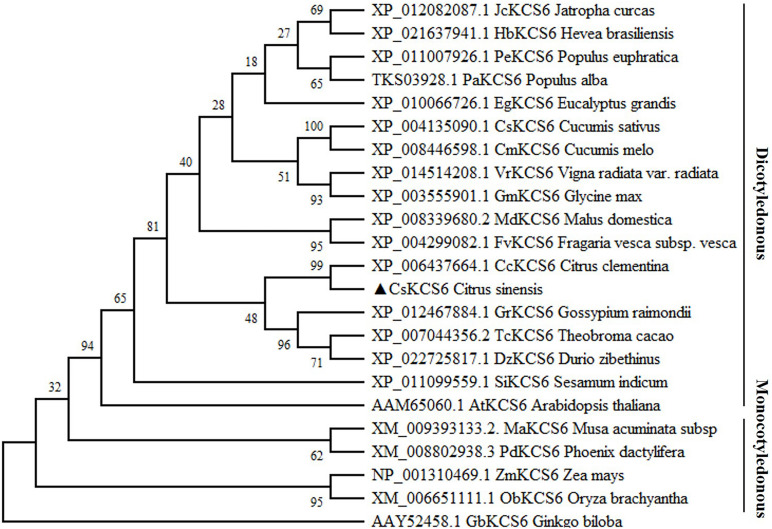
Phylogenetic tree of the homologs of CsKCS6 and other KCS proteins. The solid lines represent monocotyledonous and dicotyledonous plants.

In the past, a novel navel orange variety, named ‘Ganqi 3,’ was bred from a bud mutation of ‘Newhall’ navel orange. Further study revealed that the expression level of *CsKCS6* in ‘Ganqi 3’ fruit peels were significant lower than that in ‘Newhall’ fruit peels, indicating the *CsKCS6* might be involved in cuticular wax biosynthesis (Liu et al., 2016). Using the cDNA of the ‘Newhall Navel Orange’ leaf as a template, PCR amplification was performed using *CsKCS6* specific primers (Supplementary Table S1). Sequence analysis showed that The full-length cDNA sequence of *CsKCS6* (GenBank accession number MT259183) was 1750 bp, with an open reading frame (ORF) of 1491 bp. The CsKCS6 protein comprised 496 amino acids, with a putative molecular weight of 56.22 kDa and an isoelectric point of 9.18. Sequence analysis revealed that CsKCS6 is highly homologous to other KCS6 proteins in plants, including *Citrus clementina* CcKCS6 (98%), *Hevea brasiliensis* HbKCS6 (67%), *Jatropha curcas* JcKCS6 (68%), *Glycine max* GmKCS6 (67%), *Malus domestica* MdKCS6 (69%), *Populus euphratica* PeKCS6 (68%), *Theobroma cacao* TcKCS6 (70%), and *Sesamum indicum* SiKCS6 (67%). Structural analysis revealed that the FAE1-CUT1-RppA conserved domain was found at amino acid positions of 80–368 in CsKCS6 and that the ACP_syn_III_C conserved domain was found at 384–466. Both major domains belong to the thiolase-like superfamily. Among them, the Cys residue at amino acid position of 225 is the catalytic active site of the condensing enzyme and is highly conserved among different species (Figure 1). In this study, we used the maximum likelihood method of MEGA 6 software to construct a phylogenetic tree of plant KCS6 proteins. The results showed that the CsKCS6 protein formed a large branch with the KCS6 proteins of dicotyledonous plant species such as *Citrus clementina*, *Malus domestica*, *Gossypium raimondii*, and *Glycine max*. The CsKCS6 protein was most closely related to the clementine CcKCS6 and clustered onto the same small branch. In addition, the KCS6 protein of the monocotyledonous plant species *Musa acuminata*, *Zea mays*, *Oryza brachyantha*, and *Phoenix dactylifera* was classified as another large branch, and this protein was the most related to CsKCS6. The evolution of these protein sequences is essentially consistent with the process of plant evolution in nature (Figure 2).

### Analysis of the *CsKCS6* Expression Pattern in Navel Orange

**FIGURE 3:**
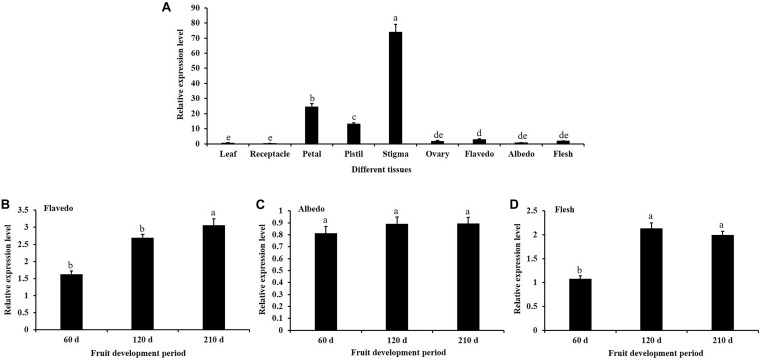
Expression analysis of *CsKCS6* in Newhall navel orange. **(A)** Spatial-specific expression of *CsKCS6* in different organs. **(B–D)** Expression pattern of *CsKCS6* in flavedo, albedo and flesh tissues during the fruit development period. The y-axis shows the relative gene expression levels, as calculated by the 2^–ΔΔCT^ method, in which *CsACTIN* served as the endogenous reference. Data are shown as the means ± SE calculated from three biological replicates. Different small letters represent significant differences at *P* < 0.05 according to Duncan’s multiple range tests.

The spatiotemporal expression of *CsKCS6* in Newhall navel orange was determined via qPCR. The results showed that *CsKCS6* Significantly high expression in the stigmas, followed by the petals and pistils. But substantially lower expression in tissues such as flavedo, albedo, flesh, leaf, receptacle, and ovary (Figure 3A). The expression level of *CsKCS6* in the flavedo and flesh of Newhall navel orange increased with fruit development. However, no significant changes were observed in the albedo of Newhall navel orange during fruit development (Figures 3B–D).

**FIGURE 4:**
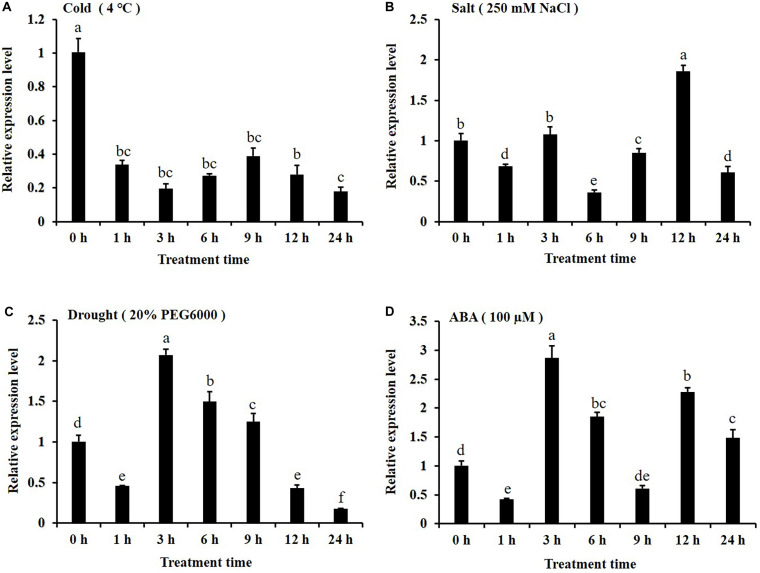
Expression of *CsKCS6* in the leaves of Newhall navel orange plants under abiotic stress according to qRT-PCR **(A–D)** after cold (4°C), salt, drought and ABA treatments. The y-axis shows the relative gene expression levels, as calculated by the 2^–ΔΔCT^ method, in which *CsACTIN* served as the endogenous reference. Data are shown as the means ± SE calculated from three biological replicates. Different small letters represent significant differences at *P* < 0.05 according to Duncan’s multiple range tests.

In this study, the expression patterns of *CsKCS6* gene in leaves of ‘Newhall’ navel orange under abiotic stress were investigated by qPCR. After 4°C treatment, the expression level of *CsKCS6* declined significantly at 1 h (about threefold lower than 0 h), remained this level from 1 to 12 h, and then decreased again at 24 h (Figure 4A). Under the 250 mM NaCl stress, the expression level of *CsKCS6* decreased significantly at 6 h, increased at 3 h, declined again at 6 h, increased to the maximum value (about 1.5-fold higher than 0 h) at 12 h, and then decreased significantly at 24 h (Figure 4B). After simulated drought treatment with 20% PEG6000, the *CsKCS6* transcript decreased significantly at 1 h, increased to the maximum value (about twofold higher than 0 h) at 3 h and then decreased gradually from 3 to 24 h (Figure 4C). After 100 μM ABA treatment, the expression level of *CsKCS6* decreased significantly at 1 h, peaked at 3 h (about 2.8-fold higher than 0 h), decreased again from 3 to 9 h. Surprisingly, the *CsKCS6* expression increased again to a high level (about 1.5-fold higher than 0 h) at 12 h after ABA treatment, but finally declined at 24 h (Figure 4D).

### Phenotypic Analysis of Transgenic *Arabidopsis* Overexpressing *CsKCS6*

**FIGURE 5:**
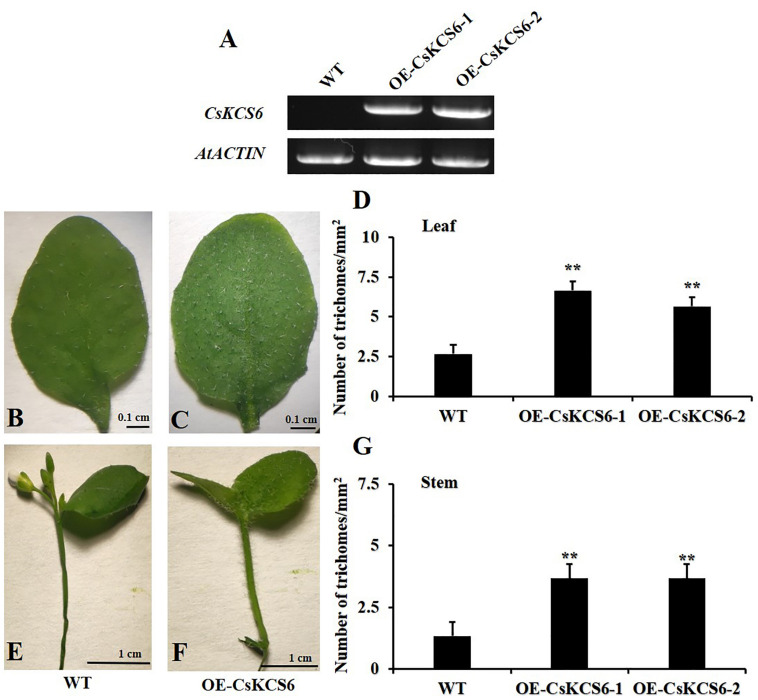
Phenotypes of the WT and two transgenic *Arabidopsis* lines (OE-KCS6-1, OE-KCS6-2). **(A)** Expression of *CsKCS6* in different leaves of the WT and transgenic *Arabidopsis* lines. *AtACTIN* was used as a reference gene. **(B)** Leaves of the WT and **(C)** transgenic lines of 6-week-old. **(D)** The trichome densities in leaves of two *Arabidopsis* genotypes show in **(B,C)**. **(E)** Stems of the WT and **(F)** transgenic lines. **(G)** The trichome densities in stems of two *Arabidopsis* genotypes show in **(E,F)**. The data are shown as the means ± SE of three biological replicates. The asterisks indicate that the values of the transgenic *Arabidopsis* line are significantly different from the values of the WT (^∗^*P* < 0.05; ^∗∗^*P* < 0.01).

To investigate the function of *CsKCS6* in plants, we introduced a 35S:*CsKCS6*-pCAMBIA1301 vector into *Agrobacterium tumefaciens* strain LBA4404, which were then transformed into *Arabidopsis* plants. The T_0_ generation seeds were screened on MS medium containing 10 mg/L hygromycin to obtain nine resistant seedlings. PCR detection was performed using specific primers of *CsKCS6* and *Hyg* (Supplementary Table S1-P4, P7) after transplanting, and five positive plants were identified (Supplementary Figure S1). The cDNA was derived from the leaves of T_2_ generation *Arabidopsis* plants. Semi-quantitative PCR detection showed that *CsKCS6* was strongly expressed in the leaves of five transgenic lines, while *CsKCS6* was not detected in WT. Therefore, OE-CsKCS6-1 and OE-CsKCS6-2 were used for further research (Figure 5A).

Observing the performance of transgenic Arabidopsis seedlings and wild-type seedlings of the same age, it is found that the trichome density on the surface of the leaves and stems of the transgenic plants is higher than that of the wild-type plants, and the transgenic leaves and stems of the plants are rougher than the wild-type plants (Figures 5B,C,E,F). Subsequently, we obtained photos of transgenic and wild-type *Arabidopsis thaliana* through stereomicroscopes, and then statistical analysis based on the pictures showed that the number of trichomes per square millimeter on the transgenic *Arabidopsis* leaves was more than twofold that of the WT, forming a very significant difference (Figure 5D). The number of trichomes on the stems of transgenic *Arabidopsis* was also significantly higher than that of the WT, with a difference of about threefold (Figure 5G).

### Cuticular Wax Morphology and Composition of Wax Present on Leaves of the WT and Transgenic *Arabidopsis* Plants

**FIGURE 6:**
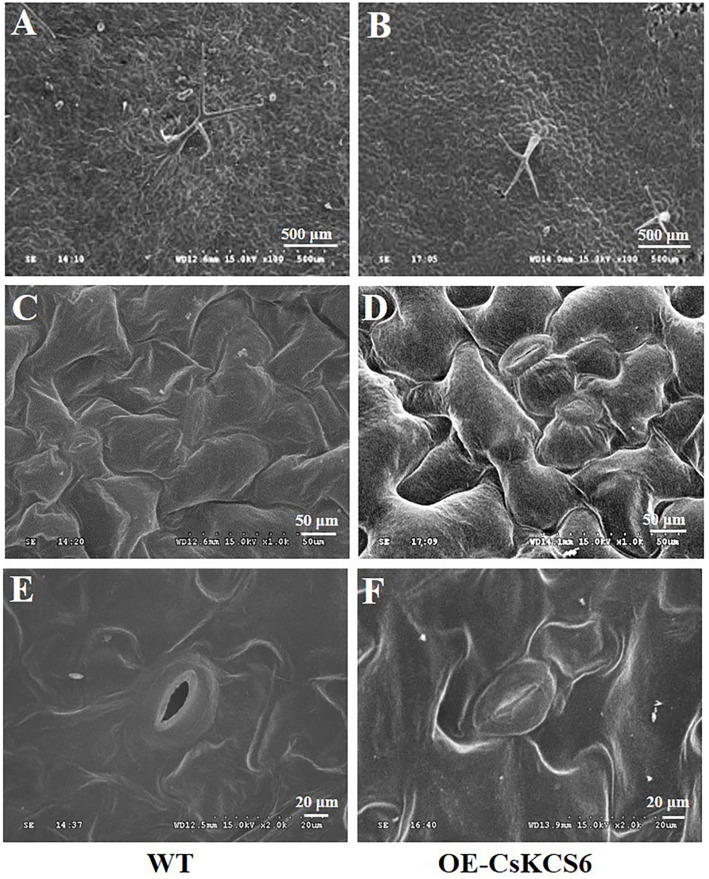
Morphology of the wax of the leaf surfaces of WT and transgenic plants. **(A)** Wax on the trichomes of WT and **(B)** transgenic plants (100×, Bar = 500 μm). **(C)** Wax on the adaxial side of the leaves of the WT and **(D)** transgenic plants (1000×, Bar = 50 μm). **(E)** Wax on the abaxial side of the leaves of WT and **(F)** transgenic plants (2000×, Bar = 20 μm).

Scanning electron microscopy was used to observe the morphology of the cuticular wax present on the leaf surfaces of the WT and transgenic *Arabidopsis*. The results showed that the trichomes on the leaf surfaces of the transgenic lines were more abundant than those of the WT (Figures 6A,B), this phenotypic difference is consistent with the statistics of trichomes in Figure 5D. However, no significant difference was detected in the morphology of the cuticular wax between the adaxial and abaxial sides of the leaves of the WT and transgenic *Arabidopsis* plants (Figures 6C–F).

**FIGURE 7:**
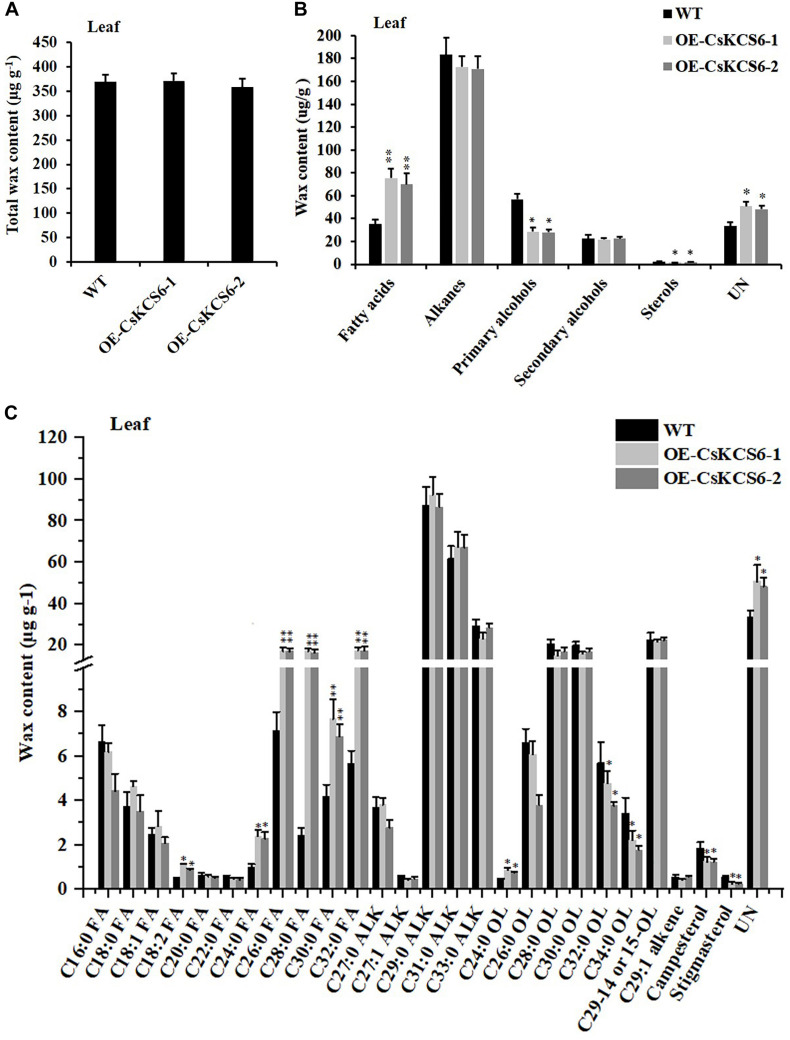
Cuticular wax content and composition of leaves of WT and transgenic *Arabidopsis* plants. **(A)** Total wax content, **(B)** wax composition, and **(C)** identified waxes in the leaves of the WT and two transgenic plants. FA, fatty acids; ALK, alkanes; OL, alcohols; UN, unknown component. The values are means ± SE of three biological replicates, and the asterisks indicate significant differences between WT plants and transgenic lines (*P < 0.05; **P < 0.01).

GC–MS analysis revealed that the total wax content of the two transgenic lines was similar to that of the WT (Figure 7A). The amount of fatty acids in the transgenic lines was about twofold higher than that in the WT plants. However, the contents of primary alcohols and sterols in the transgenic strain lines were reduced about 2- and 1.5-fold respectively in compare with the WT. There was no significant difference in the contents of alkanes and secondary alcohols (Figure 7B). Meanwhile, the two transgenic lines had much greater amounts (increased by about 40%) of fatty acids with even chain length ≥ C_24_ compared to the WT plants. But the amounts of C_32_ and C_34_ primary alcohol reduced by 35% in the transgenic lines (Figure 7C).

### Morphology and Composition of Cuticular Wax on the Stems of WT and Transgenic *Arabidopsis* Plants

**FIGURE 8:**
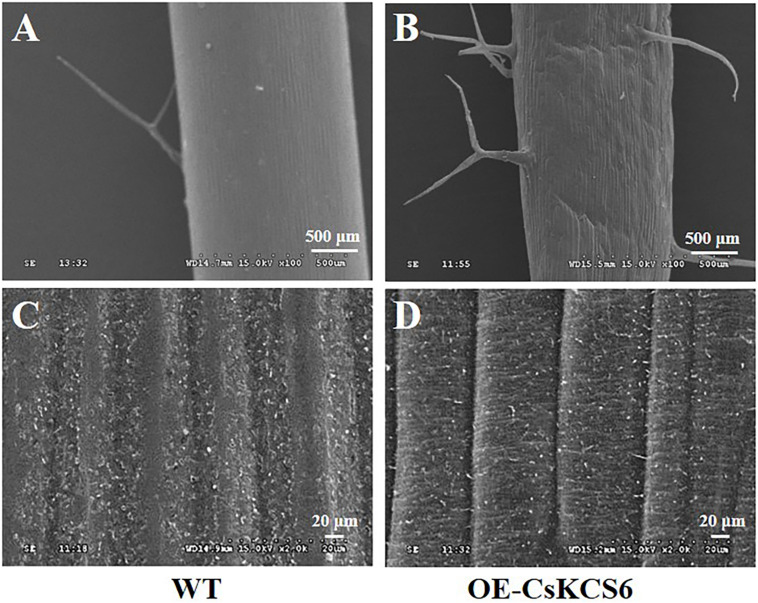
Morphology of the cuticular wax on the stem surfaces of WT and transgenic *Arabidopsis*. **(A)** Trichomes of WT stems and **(B)** trichomes of transgenic *Arabidopsis* stems (100×, Bar = 500 μm). **(C)** Wax on the surfaces of the stems of WT and **(D)** transgenic *Arabidopsis* stems (2000×, Bar = 20 μm).

Scanning electron microscopy was used to observe the morphology of the cuticular wax on the surface of the stems of WT and transgenic *Arabidopsis* plants. The results showed that obviously more trichomes were present on the surfaces of the stems of the transgenic *Arabidopsis* plants than on those of the WT plants (Figures 8A,B), this phenotypic difference is consistent with the statistics of trichomes in Figure 5G. There were many granular wax crystals deposited on the surface of the stems of both genotypes (Figures 8C,D). In addition, many shallow ridges were detected on the stem surfaces of the transgenic *Arabidopsis* plants, which were not observed on the leaf surfaces of the WT plants.

**FIGURE 9:**
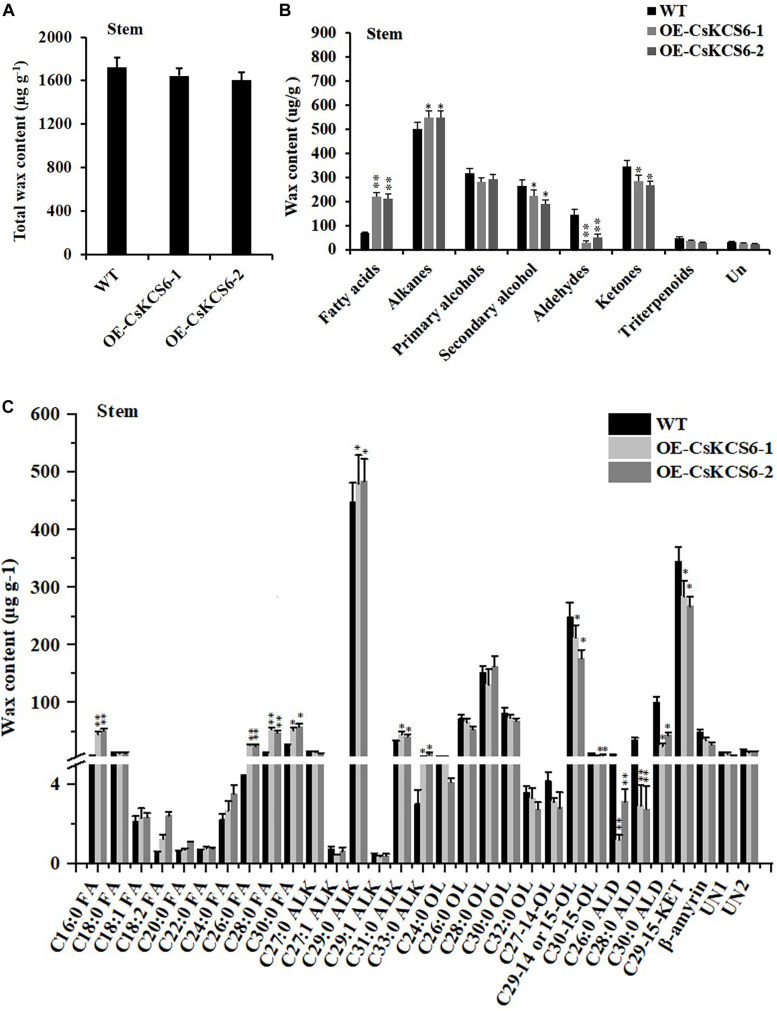
Content and composition of the cuticular wax of the stems of WT and transgenic *Arabidopsis* plants. **(A)** Wax content, **(B)** wax composition, and **(C)** identified waxes in the stems of the WT and two transgenic plants. FA, fatty acids; ALK, alkanes; OL, alcohols; ALD, aldehydes; KET, ketones; UN, unknown component. The values are the means ± SE of three biological replicates, and the asterisks indicate significant differences between the WT and transgenic lines (*P < 0.05; **P < 0.05).

GC–MS analysis revealed that the total wax content on the stem surfaces of the two transgenic lines was similar to that of the WT (Figure 9A). The total amount of fatty acids was increased by more than twofold compared with the WT plants. The alkanes amount in the transgenic lines increased by only about 18% in comparison to the WT plants. In contrast, the amounts of primary alcohols, aldehydes, and ketones decreased significantly in the transgenic lines. The most drastic decline was observed in aldehydes, which reduced by more than 30% in the transgenic lines (Figure 9B). Notably, the increase in fatty acids content was mainly observed in even chain length chains of C_16_ and C_26__–__30_. And the decrease of aldehydes was mainly attribute to the reduction of C_2__6_, C_28_, and C_30_ aldehyde (Figure 9C).

### Analysis of Cuticular Permeability of the Leaves

**FIGURE 10:**
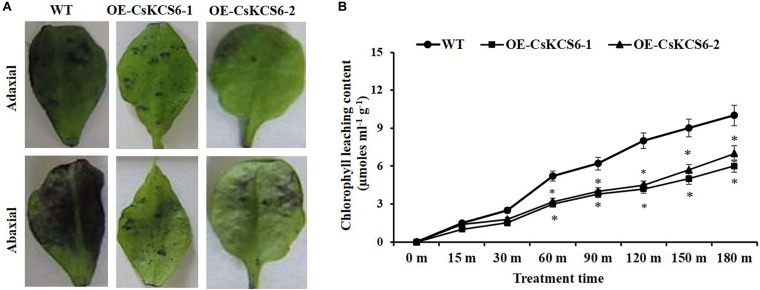
Alterations in cuticle permeability of *CsKCS6* transgenic *Arabidopsis*. **(A)** TB staining, **(B)** chlorophyll leaching content of the WT and transgenic *Arabidopsis* plants after alcohol treatment.

To explore whether the epidermal permeability was altered in *CsKCS6* transgenic lines, the epidermis of leaves collected from the CsKCS6 transgenic lines and WT was stained with TB, and the chlorophyll leaching contents was measured under alcohol treatment. The results showed that both sides of the leaves of the WT were stained much deeply than were the transgenic lines (Figure 10A). Furthermore, the chlorophyll leaching contents of the WT and transgenic lines continuously increased after the alcohol treatment; however, the chlorophyll leaching contents of the transgenic lines was lower than that of the WT at all the time points of the alcohol treatment, and the difference was significant after 60 min, and the chlorophyll leaching content extracted was only about half of that of the WT at 180 min (Figure 10B).

### Ectopic Expression of *CsKCS6* in *Arabidopsis* Enhanced Drought Tolerance

**FIGURE 11:**
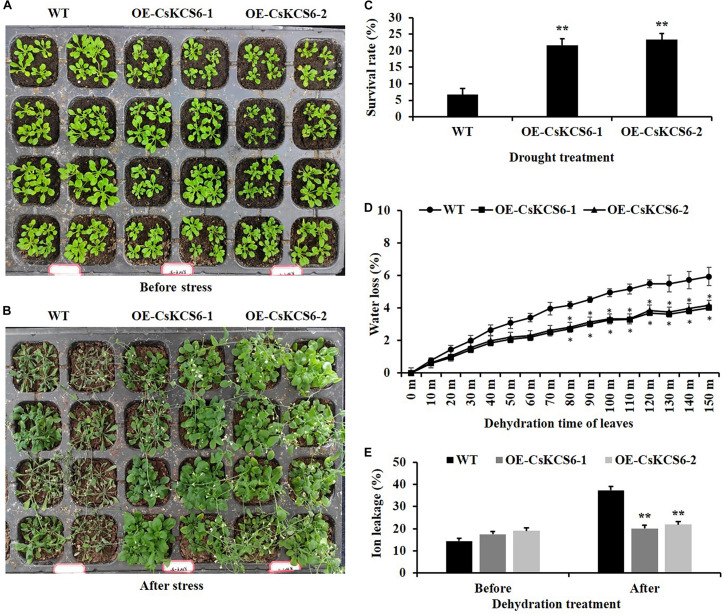
Phenotypes and physiological traits of WT and transgenic *Arabidopsis* lines after drought treatments. **(A,B)** Phenotypes of two genotypes after drought treatment, as well as their **(C)** survival rate, **(D)** water loss rate, and **(E)** ion leakage. Leaves of 6-week-old WT and transgenic plants were collected for measuring the physiological traits. The data are shown as the means ± SE of three biological replicates. The asterisks indicate that the values of the transgenic *Arabidopsis* line are significantly different from the values of the WT (*P < 0.05; **P < 0.01).

To study whether *CsKCS6* is involved in the response to osmotic stress, the plant phenotype, survival, water loss rate and ion leakage of the WT and two transgenic lines were analyzed. After drought treatment for 10 days and rewatering for 3 days, the leaf wilting of the WT plants was more severe than that of the two transgenic lines (Figures 11A,B). Moreover, the survival percentages of OE-KCS6-1 and OE-KCS6-2 were 21.66 and 23.33%, respectively, which were significantly higher than the survival rate of the WT plants (6.66%) after drought treatment (Figure 11C).

In addition, the transpiration rates of the leaves of the transgenic *Arabidopsis* plants were significantly lower than those of WT plants from 80 to 150 min after dehydration (Figure 11D). Compared with that of the WT plants, the ion leakage in the leaves of the transgenic *Arabidopsis* plants was also obviously reduced after the dehydration treatment. These results indicate that ectopic expression of *CsKCS6* can reduce the cell membrane permeability of *Arabidopsis* leaves (Figure 11E). Taken together, these results suggest that ectopic expression of *CsKCS6* can improve the drought tolerance of transgenic *Arabidopsis*.

### Ectopic Expression of CsKCS6 in *Arabidopsis* Enhanced Salt Tolerance

**FIGURE 12:**
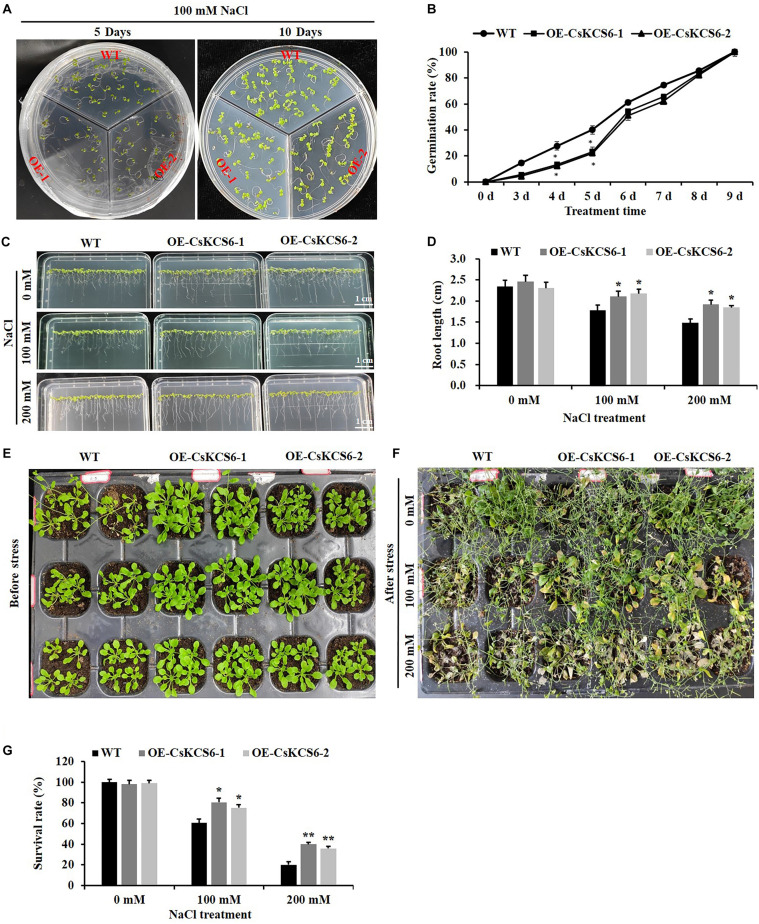
Phenotypes and physiological traits of the WT and transgenic *Arabidopsis* lines after salt treatment. **(A)** Seed germination. T_2_ and WT seeds were sown on MS media, with 30 seeds sown per line. **(B)** Germination rates calculated from **(A)**, **(C)** root growth under salt stress treatment. **(D)** Root length (root length ≥ 5 mm) calculated from **(C)**, **(E,F)** phenotypes of two genotype lines after salt treatment. **(G)** Survival. The data are shown as the mean ± SE of three biological replicates. The asterisks indicate significant differences between transgenic *Arabidopsis* lines and WT (*P < 0.05; **P < 0.01).

To analyze the role of *CsKCS6* in the plant response to salt stress, we investigated the effects of salt stress on the seed germination and root length of the WT and two transgenic lines. The seed germination rate of the two transgenic *Arabidopsis* lines was significantly lower than that of the WT within the first 5 days of 100 mM NaCl treatment. Interestingly, the germination rate of the two transgenic lines was similar to that of the WT plants after 8 days of 100 mM NaCl treatment (Figures 12A,B).

Statistical analysis showed that there was no significant difference in root length between the 2-week-old transgenic and wild-type Arabidopsis, and both could reach about 2.4 cm. However, after 100 and 200 mM NaCl treatment, the root length of the transgenic lines was about 2.2 and 1.8 cm respectively, which was significantly higher than that of the WT plants (about 1.7 and1.4 cm) (Figures 12C,D).

In addition, we compared the phenotypic differences between the WT and two transgenic lines after salt stress treatment, and more severe damage was observed in the WT plants than in the two transgenic lines after the 100 and 200 mM NaCl treatments (Figures 12E,F). Furthermore, after 100 mM NaCl treatment, the survival rate of wild-type Arabidopsis is only 60%, while the survival rate of transgenic lines reaches about 80%. When the concentration of NaCl treatment reaches 200 mM, the survival rate of the transgenic lines (40%) was about twofold higher than that of the WT (20%) (Figure 12G).

## Discussion

Very-long-chain fatty acids are biosynthesized from the extension of C_16_ or C_18_ fatty acids, and the extension is catalyzed by the multienzyme complex FAE; thus, FAE components are direct precursors of cuticular wax biosynthesis. The first step of VLCFA biosynthesis is to produce β-ketoacyl-CoA, which is catalyzed by KCS (Kunst and Samuels, 2003; Yeats and Rose, 2013).

Members of the KCS family are often described as 3-ketoacyl-CoA synthases, type III polyketide synthases and FAEs, while the characteristic regions of the KCS6 protein usually contain active site residues and motifs involved in substrate binding (Funa et al., 2002). In this study, multiple sequence alignment revealed that the amino acid sequence of CsKCS6 contained two conserved domains, FAE1-CUT1-RppA and ACP_syn_III_C, which belong to the thiolase-like superfamily. The Cys residue at amino acid position of 225 is the catalytic active site of the condensing enzyme and is highly conserved among different plant species (Figure 1). These conserved sites are also present in KCS family proteins from other plant species. In addition, phylogenetic analysis revealed that CsKCS6 clustered with other KCS6 proteins from dicotyledonous plant species, especially CcKCS6, which comes from *Citrus clementine* and is the closest homolog of CsKCS6 (Figure 2). The structural features and phylogenetic results of this protein suggested that CsKCS6 belongs to the KCS gene family and might be involved in regulating the synthesis of VLCFAs in citrus.

The expression patterns of KCS family genes have been reported in many studies. For example, tissue-specific expression analysis of 21 KCS family genes in *Arabidopsis* showed that *AtKCS21*, *AtKCS7* and *AtKCS15* were underexpressed in *Arabidopsis* tissues; that *AtKCS18/AtFAE1* and *AtKCS19* were preferentially expressed in the seeds; that *AtKCS10/AtFDH*, *AtKCS4*, *AtKCS9*, *AtKCS11*, *AtKCS20*, *AtKCS2/AtDAISY*, and *AtKCS1* are widely expressed in various tissues of *Arabidopsis*; and that these proteins may be involved in VLCFA synthesis required for growth and development (Suh et al., 2005). However, *AtKCS17* is expressed only in the flowers and siliques (Joubès et al., 2008). It has been reported that *AtKCS6* is highly expressed in the flowers and siliques but not in the roots (Costaglioli et al., 2005; Suh et al., 2005). In agreement with the information in these reports, *CsKCS6* is highly expressed in the flowers of navel orange, especially in the stigmas (Figure 3). In our previous study, we reported that the amounts of VLCFAs continuously increased in the epicuticular wax of Newhall navel orange during fruit development. Accordingly, the expression of *CsKCS6* in the flavedo and albedo of Newhall navel orange also increased during fruit development (Figure 3). Thus, *CsKCS6* might be involved in the biosynthesis of VLCFAs for the cuticular wax of navel orange (Liu et al., 2015).

Cuticular wax synthesis in plants is induced by various environmental factors, such as light, water deficit and low temperature (Shepherd and Wynne Griffiths, 2006). The expression of many cuticular wax genes, including KCS family genes, also shifted under abiotic stress. For example, the expression levels of *Arabidopsis AtKCS1* and *AtKCS3* were downregulated under darkness and low temperature, and the expression of *AtKCS10* decreased under various kinds of osmotic stress (Joubès et al., 2008). In the present study, the expression level of *CsKCS6* significantly declined under low temperature stress and obviously increased at several time points after high salt, drought and ABA treatment (Figure 4). This result was in agreement with the report of Joubès et al. (2008), which indicated that the expression level of Arabidopsis *AtKCS6* was decreased under low temperature stress but increased after high salt and drought treatment. Similar result also reported by Hooker et al. (2002), which suggested that osmotic stress and ABA enhance the transcription of *AtKCS6/AtCER6* (Hooker et al., 2002). Interestingly, the expression levels of *CsKCS6* were fluctuated after high salt, drought and ABA treatments (Figure 4). As reviewed by Grundy et al. (2015), the circadian clock controls expression of a large number of genes that are responsive to abiotic stress. Thus, many abiotic stress-responsive genes were found to be expressed rhythmically under diurnal light–dark or temperature cycles. Furthermore, rhythmic expression of abiotic stress-responsive genes under constant conditions was also reported for many plant species, such as Arabidopsis, soybean and barley (Covington et al., 2008; Habte et al., 2014; Marcolino-Gomes et al., 2014). It has been proved that dark can suppress the expression of Arabidopsis *AtKCS6* (Hooker et al., 2002; Joubès et al., 2008). Thus, the *CsKCS6* transcript was probably under the control of circadian clock, leading to the fluctuated changes in *CsKCS6* expression after high salt, drought and ABA treatments.

To further study whether *CsKCS6* participates in the biosynthesis of VLCFAs for cuticular wax and regulates the osmotic stress tolerance of navel orange, transgenic *Arabidopsis* lines ectopic expression *CsKCS6* were generated in this study. Phenotypic observations and SEM analysis revealed that the density of trichomes on the leaves and stems of transgenic *Arabidopsis* was significantly higher than that of the WT plants (Figures 5, 6, 8), suggesting *CsKCS6* might be involved in the development of trichomes. To our surprise, although AtKCS16 is proved to catalyze the elongation of C_34_ to C_38_ acyl-CoAs in Arabidopsis leaf trichomes (Hegebarth et al., 2017), none of the *KCS6* homologous genes and other KCS family genes were reported to be related to trichome formation (Fiebig et al., 2000; Hooker et al., 2002; Serra et al., 2009; Weidenbach et al., 2015). The divergence between our result and previous reports is probably attributed to the gene function difference among plant species. Trichomes play an important role in plant resistance to biotic and abiotic stresses (Choi, 2010; Kim et al., 2012). Thus, the increase in trichome numbers in the transgenic *Arabidopsis* plants might enhance their tolerance to abiotic stresses.

*Arabidopsis AtCER6* is also named *AtCUT1* and *AtKCS6* (Fiebig et al., 2000). In *cer6* mutants of *Arabidopsis*, wax crystals are nearly undetectable in the stems and siliques due to the loss of *AtCER6* function (Millar et al., 1999; Fiebig et al., 2000). Moreover, overexpression of *AtCER6/AtKCS6* in *Arabidopsis* leads to waxless phenotype in stems of transgenic lines (Millar et al., 1999). Surprisingly, in the present study, no significant differences in the structure or total amount of wax were observed on the surfaces of the leaves and stems between the WT and transgenic *Arabidopsis* plants. Similar to our result, although high levels of *AtCER6*/*AtKCS6* expression were observed in the hemizygous 35S-*AtCER6*/*AtKCS6* plants, they did not exhibit higher wax amounts than those in WT plants (Millar et al., 1999). The total amount of cuticular wax in tuber periderms of *StKCS6*-silenced lines was also not significantly different from the wild-type potato (Serra et al., 2009). KCSs catalyze the rate-limiting step in VLCFA elongation and have substrate specificity (Joubès et al., 2008). Arabidopsis *AtKCS6* catalyzes the elongation of C_24_ and C_26_ VLCFAs (Jenks et al., 1994; Millar et al., 1999). Tomato *LeCER6* and Potato *StKCS6* are both involved in the biosynthesis of VLCFAs beyond C_28_ (Leide et al., 2007; Serra et al., 2009). Cotton *GhCER6* is responsible for the formation of C_26_ VLCFA (Qin et al., 2007). In agree with these reports, the amounts of VLCFAs with an even-numbered carbon chain length greater than that of C_24_ in the leaves and greater than that of C_26_ in the stems of the two transgenic lines were significantly higher than those in the WT plants, suggesting *CsKCS6* is involved in the biosynthesis of VLCFAs with chain length beyond C_24_. In addition to the striking differences in the accumulation of VLCFAs, ectopic expression of *CsKCS6* also decreased the amounts of primary and secondary alcohols, aldehydes and ketones, especially primary alcohols with chain length beyond C_28_ in leaves (decreased by 30%) and aldehydes with chain length longer than C_26_ in stems (decreased by 50%) (Figures 7, 9). The significant decreases of primary alcohols and aldehydes in transgenic plants suggested that either some wax biosynthetic genes might be suppressed by ectopic expression of *CsKCS6* or that a large block in VLCFAs entering the wax biosynthetic pathway might be occurred in transgenic plants. The latter hypothesis was supported by Von Wettstein-Knowles (1979), who suggested that VLCFA channeling was probably occurred in plants so that the substrates destined for specific wax components were not freely changed.

The permeability of the cuticle plays an important role in the adaptation of plants to osmotic stress, which affects the water retention capacity of plants and delays the damage of plants from environmental stress (Yeats and Rose, 2013). Recently, several *KCS* family genes from different plant species were reported to play an important role in regulating cuticle permeability and plant tolerance to abiotic stress. For example, overexpression of *AhKCS1* from a drought tolerant groundnut reduced the cuticle permeability and enhanced the drought tolerance in a susceptible genotype by increasing cuticular wax load in leaves (Lokesh et al., 2019). Overexpression of *BnKCS1-1* and *BnKCS1-2* in *Brassica napus* also increased drought tolerance in transgenic plants by promoting cuticular wax production (Wang et al., 2020). As reviewed by Singh et al. (2018), a number of other genes related to wax biosynthesis also have the function to enhance the resistance to abiotic stress by altering wax composition, such as *WIN1/SHN1*, *DWA1*, *MYB96*, *CER1*, *CER3/WAX2*, *FAR* family genes and so on. In agreement with these reports, a significant decrease in leaf chlorophyll permeability, TB staining, water loss rate, ion leakage and an obvious increase in the root length and survival rate were observed in the transgenic plants under drought and high salt stress (Figures 10–12), indicating that ectopic expression of *CsKCS6* can reduce the cuticle permeability and enhance plant tolerance to drought and high salt. The mechanism of *KCS6*-like genes to influence cuticle permeability in tomato (Vogg et al., 2004; Leide et al., 2007) and potato (Serra et al., 2009) is well-established. In detail, for the tomato *lecer6* mutant deficiency in *KCS6*, a decline in aliphatic components such as alkanes with chain length beyond C_30_ and an increase in cyclic triterpenoids lead to the increase in cuticle permeability (Vogg et al., 2004; Leide et al., 2007). Serra et al. (2009) reported that both the significant decrease in aliphatic compounds with chain length beyond C_28_ and total wax coverage in tuber periderm of *StKCS6*-silenced potato should be responsible for the increase of cuticle permeability. Since the wax morphology and the total wax load in the transgenic lines were similar to the WT plants, it is reasonable to deduce that the dramatic increase (about twofold) in VLCFAs with chain length beyond C_24_ in leaves of transgenic plants might be the major reason for the decrease in cuticle permeability. This result further supports the suggestion that cuticular wax composition rather than total wax load predominantly affects the cuticle permeability in plants (Leide et al., 2007; Isaacson et al., 2009; Parsons et al., 2012).

## Conclusion

In summary, in the present study, the KCS family gene *CsKCS6*, which is mainly highly expressed in flowers of Newhall navel orange, was cloned from this species. Drought, salt, and ABA treatment could increase the transcript levels of *CsKCS6* in leaves of Newhall navel orange. Ectopic expression of *CsKCS6* in *Arabidopsis* promoted a significant increase in the total VLCFA content of the leaves and stems but resulted in no significant change in total wax content. Further study revealed that the drought tolerance and salt tolerance of the transgenic *Arabidopsis* lines were significantly enhanced compared with those of the WT plants. Our results indicate that *CsKCS6* can enhance plant tolerance to drought and high salt stress by regulating the biosynthesis of VLCFAs.

## Data Availability Statement

The datasets presented in this study can be found in online repositories. The names of the repository/repositories and accession number(s) can be found in the article/ Supplementary Material.

## Author Contributions

QW, WG, and DL originally formulated the idea. LY, WH, and YL developed methodology. WG performed statistical analyses and wrote the manuscript. All authors edited and approved the final manuscript.

## Conflict of Interest

The authors declare that the research was conducted in the absence of any commercial or financial relationships that could be construed as a potential conflict of interest.

## Footnotes

1 http://citrus.hzau.edu.cn/orange/

## Supplementary Material

Supplementary Figure S1 Identification of positive transgenic plants by PCR. **(A)** The T_1_ generation transgenic lines of *Arabidopsis* were detected by specific primers of *CsKCS6* and *Hyg* genes to obtain positive plants. **(B)** Semi-quantitative detection of the expression level of transgenic *Arabidopsis*.

Supplementary Table S1 Primer sequences used for *CsKCS6* cloning, expression analysis, vector construction, and transgenic confirmation.
