# Supplementary figures and images for: Ectopic Expression of CsKCS6 From Navel Orange Promotes the Production of Very-Long-Chain Fatty Acids (VLCFAs) and Increases the Abiotic Stress Tolerance of Arabidopsis thaliana
Source: Front Plant Sci. 2020 Oct 6;11:564656. doi: 10.3389/fpls.2020.564656 (PMC7573159; doi:10.3389/fpls.2020.564656)

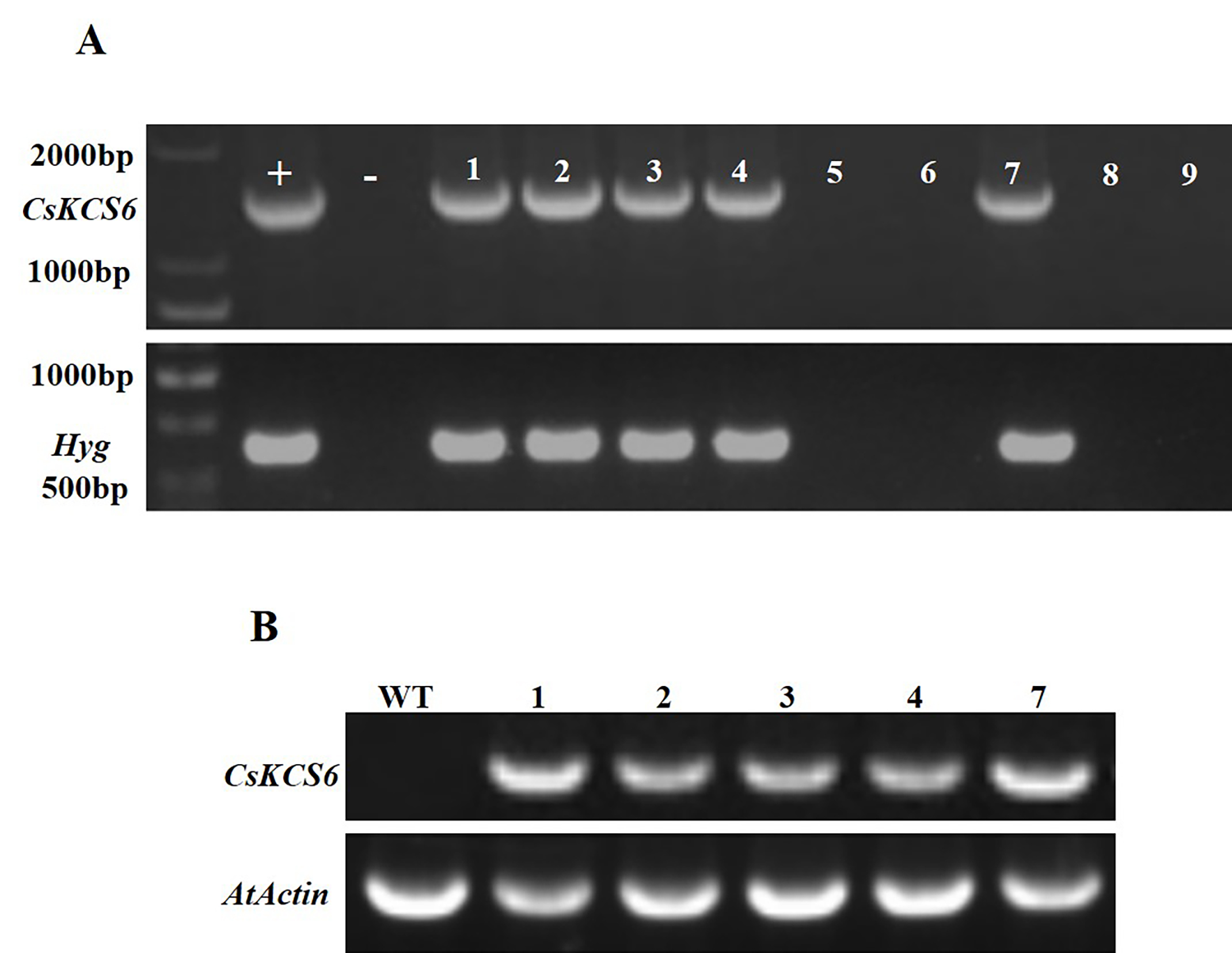

Supplement: Supplementary Figure S1 — Identification of positive transgenic plants by PCR. (A) The T1 generation transgenic lines of Arabidopsis were detected by specific primers of CsKCS6 and Hyg genes to obtain positive plants. (B) Semi-quantitative detection of the expression level of transgenic Arabidopsis. [file Image_1.jpeg]
