# Supplementary material for: Ectopic Expression of CsKCS6 From Navel Orange Promotes the Production of Very-Long-Chain Fatty Acids (VLCFAs) and Increases the Abiotic Stress Tolerance of Arabidopsis thaliana
Source: Front Plant Sci. 2020 Oct 6;11:564656. doi: 10.3389/fpls.2020.564656 (PMC7573159; doi:10.3389/fpls.2020.564656)
Supplement: Supplementary Table S1 — Primer sequences used for CsKCS6 cloning, expression analysis, vector construction, and transgenic confirmation. [file Table_1.DOC]

**Table S1. Primer sequences used for *CsKCS6* cloning, expression analysis, vector construction, and transgenic confirmation**

| **Name** | **Primer Name** | | **Sequences (5'-3')** | |
| --- | --- | --- | --- | --- |
|  |  |  | **Forward** | **Reverse** |
| *CsKCS6* cloning | | P1 | TAAATCTTCTCATCTGCCACC | AAATGAAACCGAGGAGGCTAA |
| *CsKCS6* expression | | P2 | TTCGCCACATTCATGGAGCA | ATTTCATAGGGGCCCAAGCG |
| *CsACTIN* | | P3 | ACTCATCGTACTCAGCCTTTG | TGCACCCTGTTCTTCTTACTG |
| pCAMBIA1301- *CsKCS6* construction | | P4 | GTCGATCCA ATGCCTCAAATCTTGCCTGAT  (*BamHⅠ* site is underlined) | TTCGGTACC CTAGAGCTTGACAATTTCGGG  (BstpⅠ site is underlined) |
| *CsKCS6* expression in transgenic lines | | P5 | ACCTAACAGAACTCGCCGTA | GATTGTGCGTCATCCCTTAC |
| *AtACTIN*  *Hyg* | | P6  P7 | GAAACCCTCGTAGATTGGCA  ACGGTGTCGTCCATCACAGTTTGCC | CTCTCCCGCTATGTATGTCGC  TTCCGGAAGTGCTTGACATTGGGGA |
